# MiR-361-5p inhibits colorectal and gastric cancer growth and metastasis by targeting staphylococcal nuclease domain containing-1

**DOI:** 10.18632/oncotarget.3744

**Published:** 2015-04-17

**Authors:** Fei Ma, Hongjiang Song, Baoliang Guo, Yuxin Zhang, Yasheng Zheng, Chengchun Lin, Ying Wu, Guijie Guan, Ruihua Sha, Qingxin Zhou, Dejun Wang, Xinglu Zhou, Juan Li, Xiaohui Qiu

**Affiliations:** ^1^ Department of Endoscopy, The Affiliated Cancer Hospital, Harbin Medical University, Harbin, China; ^2^ Department of Gastrointestinal Surgery, The Affiliated Cancer Hospital, Harbin Medical University, Harbin, China; ^3^ Department of General Surgery, The Second Affiliated Hospital of Harbin Medical University, Harbin, China; ^4^ Department of General Surgery, Central Hospital of Jiuzhen, China; ^5^ Department of Gastroenterology, The First Hospital of Longyan, Fujian Medical University, Longyan, China; ^6^ Department of Pathology, Hongqi Hospital, Mudanjiang Medical University, Mudanjiang, China; ^7^ Department of Digestive Disease, Hongqi Hospital, Mudanjiang Medical University, Mudanjiang, China; ^8^ Department of Ultrasound of Obstetrics and Gynecology, The First Affiliated Hospital of Harbin Medical University, Harbin, China; ^9^ Department of Medical Imaging, The Affiliated Cancer Hospital of Harbin Medical University, Harbin, China; ^10^ Department of Pathology, The Second Affiliated Hospital of Harbin Medical University, Harbin, China; ^11^ The Second Hospital of Longyan, Fujian Medical University, Longyan, China

**Keywords:** miR-361-5p, Tudor staphylococcal nuclease 1, colorectal carcinoma, gastric cancer, RNA-induced silencing complex (RISC)

## Abstract

MicroRNAs (miRs) function as key regulators of gene expression and their deregulation is associated with the carcinogenesis of various cancers. In the present study, we investigated the biological role and mechanism of miR-361-5p in colorectal carcinoma (CRC) and gastric cancer (GC). We showed that microRNA-361-5p (miR-361-5p) was down-regulated in CRC and GC in comparison to the controls. Meanwhile, the expression levels of miR-361-5p negatively correlated with lung metastasis and prognosis in clinical CRC patients. Overexpression of miR-361-5p markedly suppressed proliferation, migration and invasion of cancer cells. Additionally, this phenotype could be partially rescued by the ectopic expression of staphylococcal nuclease domain containing-1 (SND1). SND1 was identified as a target of miR-361-5p using bioinformatics analysis and *in vitro* luciferase reporter assays. In turn, SND1 bound to pre-miR-361-5p and suppressed the expression of miR-361-5p, thus exerting a feedback loop. Most interestingly, *in vivo* studies showed that restoration of miR-361-5p significantly inhibited tumor growth and especially the lung metastasis in nude mice. Therefore, it could be concluded that miR-361-5p functions as a tumor-suppressive miRNA through directly binding to SND1, highlighting its potential as a novel agent for the treatment of patients with CRC and GC.

## INTRODUCTION

Colorectal cancer (CRC) is one of the most common malignancies and among the leading causes of death in the industrialized world [1]. Although fecal occult blood test and endoscopy have contributed to a great reduction in CRC mortality and most of the CRC patients in the early stage (Dukes A and B) can successfully be treated via surgical resection, many CRC patients are still found to have tumor metastasis in the advanced stage (Dukes C and D). Advanced stage is usually refractory to existing therapies and associated with poor survival, resulting in the significant drop in the 5-year survival rates [2]. Tumor growth and metastasis are complicated biological process that involves a subset or individual cancer cells detaching from the primary tumor, migrating to the blood/lymph, and colonizing distant organs or tissues [3]. Dysregulation of signaling pathways, and abnormal expression and function of some molecules are often related to cancer metastasis [4]. Therefore, deciphering the potential mechanisms underlying cancer invasion and metastasis is of paramount importance in designing novel and effective therapeutic strategies. Identification of these new biomarkers which are sensitive and specific for CRCs, is urgently needed and will open up many avenues for diagnosis and therapeutic exploitation.

To date, microRNAs (miRNAs), a class of non-coding RNA, were found to get involved in various fundamental biological processes, such as cell proliferation, apoptosis and differentiation, and implicated in cancer development and progression [5]. Functioning as regulatory molecules, miRNAs are able to suppress gene expression by inhibiting the protein translation process and/or degrading the respective target messenger RNA [6]. Thus, to consider miRNAs as therapeutic targets in cancer is plausible. Increasing number of studies has strongly shown that deregulated expressions of several miRNAs correlate with CRC development. Therefore, identification of CRC-associated miRNA as biomarkers for early tumor detection, prognostication and treatment is of great importance. Recently, miR-361-5p emerged as an important regulator of tumorigenicity and cancer metastasis [7], however, its role in CRCs remains unknown.

Tudor staphylococcal nuclease (SND1), also known as Tudor-SN or p100, is a highly conserved and ubiquitously expressed multifunctional protein. It can regulate various intracellular processes, such as RNA interference as a nuclease in the RNA-induced silencing complex (RISC), mRNA splicing and stability, and transcription as a co-activator [8]. SND1 was first identified as a transcriptional co-activator of Epstein-Barr virus nuclear antigen 2 (EBNA2), Pim-1, polycystin-1 (PC1), STAT5, STAT6, and c-Myb (8–9). Recent studies have uncovered the role of SND1 in carcinogenesis of diverse organs [9]. It has been detected that SND1 expression increases in human tumors such as prostate, breast, colon, and hepatocellular carcinomas, and is positively related to the stages and grades of cancer. Especially, its overexpression is associated with highly aggressive metastatic disease, especially in CRCs [10]. In nude mice xenograft studies, siRNA-mediated inhibition of SND1 abrogated and overexpression of SND1 increased growth of human HCC and prostate cancer cells [11]. SND1 also augmented tumor angiogenesis by activating NF-κB which induces miR-221 and angiogenic factors Angiogenin and CXCL16 [12]. These studies indicate that SND1 promotes aggressive cancer by multiple ways. Although SND1 is known to stimulate tumor growth in multiple cancer contexts including CRCs, its upstream signal in CRCs has previously not been investigated.

In this study, we aimed to enlighten the significance of miR-361-5p in CRC and its potential target gene via a series of experiments *in vitro* and *in vivo*. We showed that miR-361-5p contributed to CRC cell proliferation and participated in the induction of metastasis of CRC by suppressing the expression of SND1. Furthermore, we demonstrated that SND1 inhibited miR-361-5p expression by directly binding to pre-miR-361-5p, subsequently leading to downregulation of mature miR-361-5p expression. Clinically, our analysis of 60 tumor samples from CRC patients revealed that low expression of miR-361-5p in CRC tissues was significantly correlated with advanced tumor stages, lung metastasis, and poor survival. Importantly, the expression level of miR-361-5p was negatively correlated with the expression of SND1. Additionally, we also extended our findings to gastric cancer. Altogether, these data not only associate miR-361-5p with growth and metastasis in CRC and GC, but also provide a new therapeutic target for treating CRC and GC.

## RESULTS

### miR-361-5p expression in human GC and CRC is decreased which is significantly correlated with poor survival

**Figure 1:**
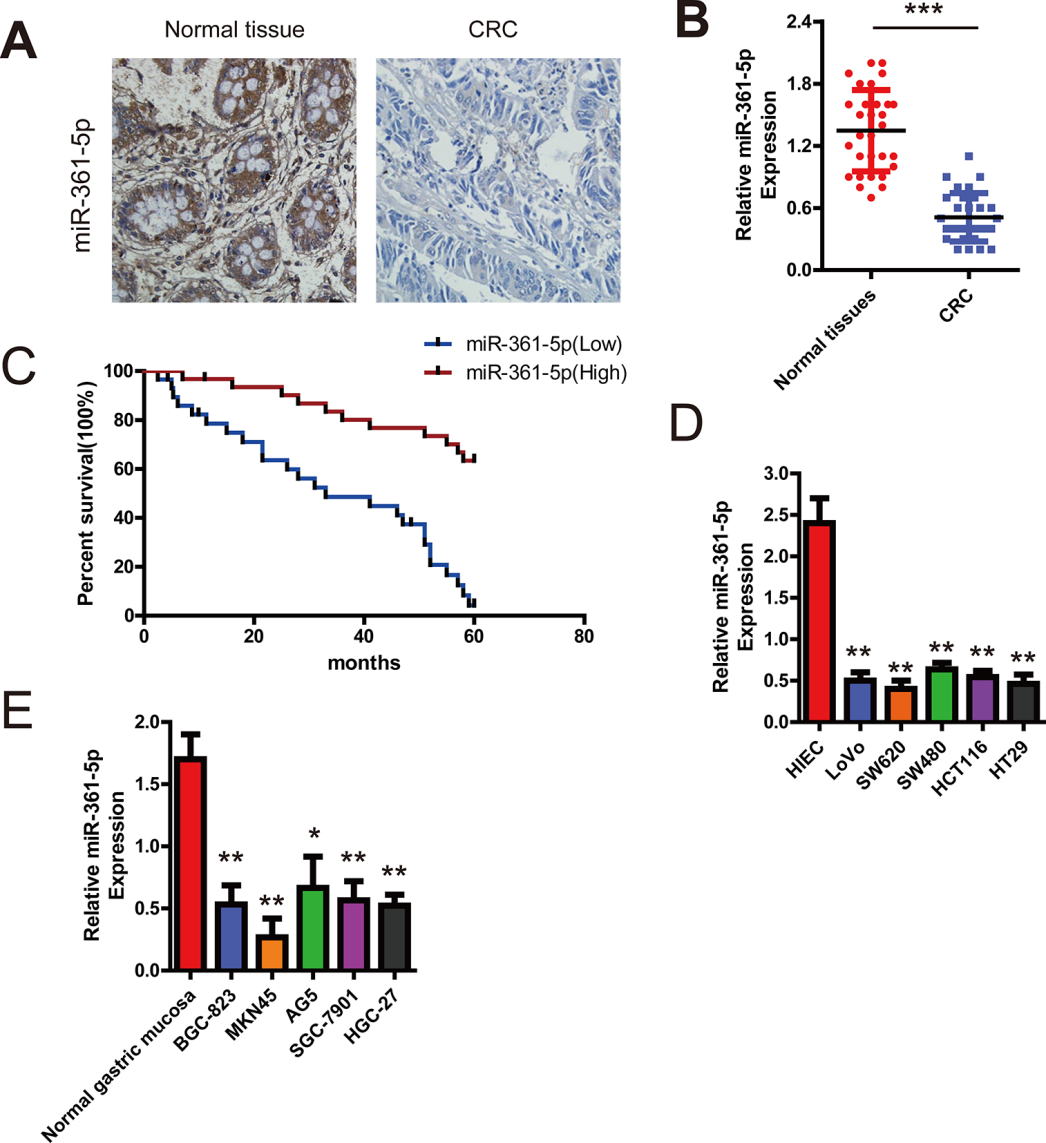
Expression of miR-361-5p and its effect on CRC patients' survival **A.** Analysis of miR-361-5p in situ hybridization (ISH) signal in CRC. Representative staining images for miR-361-5p expression in CRC tissues and non-tumor colorectal tissues are shown (200 × magnification). **B.** qRT-PCR assay showing that miR-361-5p expression is significantly decreased in a group of 60 CRC tissues when compared to matched normal tissues (*P* < 0.001). **C.** Association between miR-361-5p expression and survival of CRC patients. Kaplan-Meier survival test indicates that patients expressing low miR-361-5p levels exhibited significantly shorter survival (*P* = 0.014). **D.** Comparison of expression level of miR-361-5p between normal colorectal tissues and CRC cell lines using qRT-PCR assay. **E.** Relative expression of miR-361-5p in 5 gastric cancer cell lines compared with normal gastric mucosa using qRT-PCR assay. The results are presented as means ± SD (*n* = 3 for each panel). Statistical significance was concluded at *P < 0.05, **P < 0.01, ***P < 0.001; # represents no statistical significance.

To determine the role of miR-361-5p in the pathogenesis of CRC, we investigated miR-361-5p levels in normal colorectal and CRC tissues in a group of 60 CRC patients and various CRC cells using ISH and qRT-PCR respectively. As shown in the Figure 1A, compared with the corresponding non-tumor samples, the miR-361-5p levels was significantly decreased in 51 of 60 patients. The scatter diagram also demonstrated the decrease of miR-361-5p levels in CRC samples versus normal tissues, with an average 3.08-fold decrease (*P* = 0.004) (Figure 1B). Furthermore, Kaplan–Meier analysis showed that the survival time of patients with low miR-361-5p expression was significantly shorter (Figure 1C). The levels of miR-361-5p were also found to be significantly lower in CRC cell lines than in normal colorectal tissue (Figure 1D). Interestingly, the consistent decreased expression of miR-361-5p was demonstrated in GC compared with normal gastric mucosa (Figure 1E). Taken together, these results suggest that the miR-361-5p expression is frequently down-regulated in GC and CRC, and is correlated with poor prognosis, suggesting that miR-361-5p functions as a tumor suppressor in CRC and GC development.

### miR-361-5p suppresses cancer cell proliferation in CRC and GC

**Figure 2:**
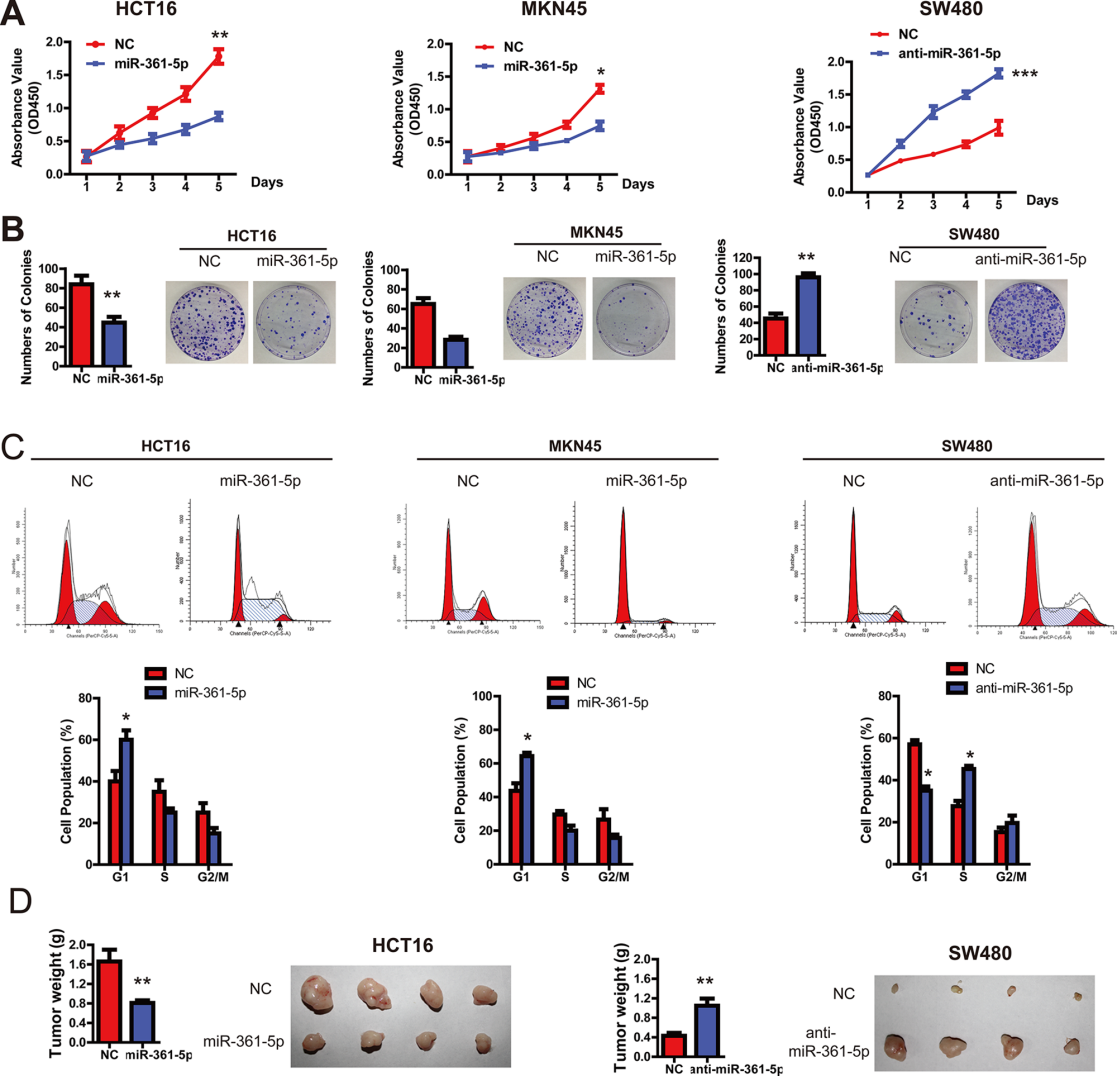
miR-361-5p suppresses CRC and GC cell growth *in vitro* and *in vivo* **A.** The CCK-8 assay was used to measure the effect of miR-361-5p on CRC and GC cell growth. **B.** The effect of miR-361-5p on colony formation in HCT116, SW480 and MKN45 cells. **C.** Flow cytometry analysis was used to analyze GC and CRC cells in G1, S, and G2/M phase. **D.** The effect of miR-361-5p on tumor formation in a nude mouse xenograft model. Nude mice (*n* = 10 per group) were injected subcutaneously into opposite flanks with 1.5 × 10^6^ control cells and miR-361-5p -overexpressed cells. The mice were sacrificed and the tumors were then removed, weighed and compared. The results are presented as means ± SD (*n* = 3 for each panel). Statistical significance was concluded at *P < 0.05, **P < 0.01, ***P < 0.001; # represents no statistical significance.

Having observed the association of miR-361-5p expression and poor survival in CRC patients, we set out to functionally characterize the effects of miR-361-5p on CRC and GC cells. Firstly, cell proliferation assays revealed that miR-361-5p overexpression significantly reduced the growth rates of HCT116 and MKN45 cells, whereas silencing miR-361-5p expression significantly promoted the proliferation of SW480 cells (Figure 2A). Colony formation assays further confirmed the anti-proliferation function of miR-361-5p in CRC and GC cells (Figure 2B). Cell-cycle assays also supported the results, because overexpression of miR-361-5p was found to be related to G1 cell-cycle arrest, which was evidenced by the reduced percentage of S and G2/M and the increased percentage of G1 (Figure 2C). Importantly, an *in vivo* tumor formation assay in a nude mouse model demonstrated that miR-361-5p overexpression significantly inhibited the tumorigenesis of CRC cells compared with the vector control (Figure 2D). Collectively, these data clearly demonstrate that miR-361-5p has a growth-suppressive function in CRC and GC.

### miR-361-5p inhibits the invasion and metastasis of CRC and GC

**Figure 3:**
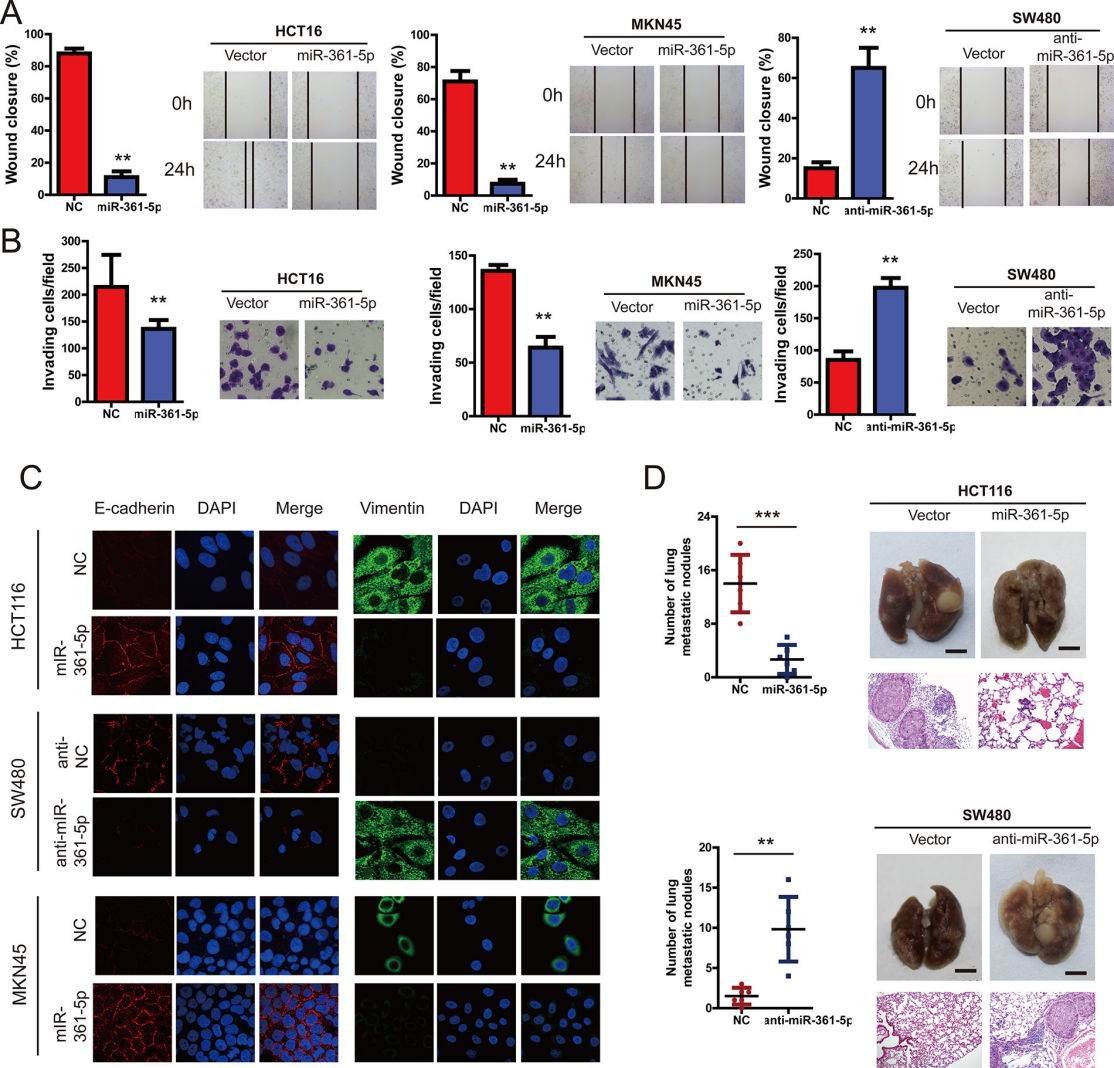
miR-361-5p suppresses invasion and metastasis of CRC and GC cells CRC and GC cell lines infected with miR-361-5p expression vector or miR-361-5p inhibitior or NC were used in the following studies. **A.** We performed the wound-healing assay to explore the effect of miR-361-5p on the motility of CRC and GC cells. The percentage of wound healing is shown in the diagrams. **B.** Effects of miR-361-5p on cell invasion in indicated cells *in vitro* using transwell assay. The diagrams were shown. **C.** Representative IF images indicated that miR-361-5p has an effect on the expression of EMT genes in CRC and GC cells. **D.** Effects of miR-361-5p over-expression on tumor metastasis of indicated cells in nude mice (*n* = 10 per group): the number of metastatic nodules in the lung (left-panel); representative morphological observation of lung metastases (right-upper panel); and histopathological observation of lung sections (right-lower panel). The results are presented as are means ± SD (*n* = 3 for each panel). Statistical significance was concluded at *P < 0.05, **P < 0.01, ***P < 0.001; # represents no statistical significance.

Next, we examined the effects of miR-361-5p on the ability of CRC and GC cells to invade and migrate. Wound-healing assays indicated that the level of miR-361-5p expression was inversely correlated with the rates of wound healing: lower levels of miR-361-5p expression with faster healing (Figure 3A). Similarly, transwell assays with Matrigel demonstrated that HCT116 and MKN45 cells overexpressing miR-361-5p were found to have significantly lower rate of invasion than control cells, whereas SW480 cells expressing anti-miR-361-5p invaded faster (Figure 3B). Interestingly, there was a marked change in the expression of hallmark EMT genes in miR-361-5p-expressing cells based on immunofluorescence staining (Figure 3C). Cells with relatively low miR-361-5p expression displayed a mesenchymal-like phenotype, and the expression of the mesenchymal marker (Vimentin) was enhanced. On the contrary, miR-361-5p overexpression strongly up-regulated the epithelial marker E-cadherin. To probe the effect of the abnormal expression of miR-361-5p on tumor metastasis *in vivo*, control and miR-361-5p-overexpressed cells were injected into nude mice via the lateral tail vein. As shown in Figure 3D, the mice injected with the miR-361-5p-overexpressed CRC cells exhibited less visible metastatic nodules in the lung than the control group. The result was confirmed by histological staining and a statistical analysis, demonstrating that miR-361-5p over-expression strongly inhibited the ability of CRC cells to form metastases in the lung. Similar results were also found in the MKN45 with miR-361-5p overexpression (data not shown). Taken together, these findings suggest that miR-361-3p strongly inhibits CRC and GC cell invasion and metastasis potentialities.

### miR-361-5p decreases SND1 expression by directly binding to its 3′-UTR

**Figure 4:**
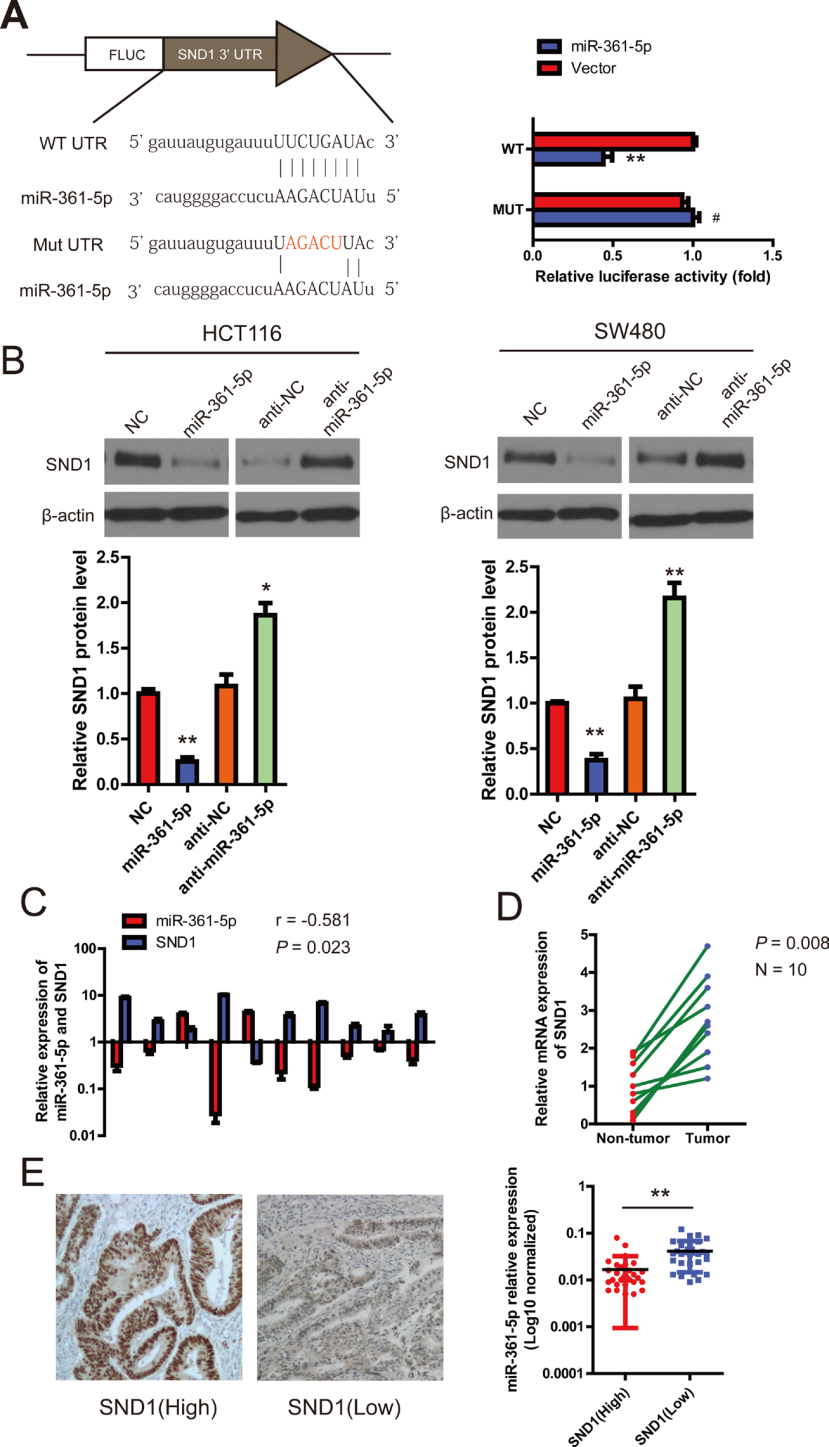
miR-361-5p downregulates SND1 expression by directly binding to its 3′-UTR **A.** Schematic representation of the luciferase reporter plasmids containing the SND1 3′-UTR and putative wild-type or mutant miR-361-5p-binding sequence in the 3′-UTR of SND1 mRNA. HEK293 cells were cotransfected with a control vector or miR-361-5p and a luciferase reporter construct containing the wild-type or mutant SND1 3′-UTR. The data were normalized and the luciferase activity of the control was set to 1. **B.** Effects of miR-361-5p dysregulation on endogenous SND1 expression which was analyzed by western blotting and RT-PCR. **C.** The correlation between miR-361-5p and SND1 mRNA expression in 10 human CRC samples using quantitative PCR. Spearman's correlation was analyzed. **D.** RT-PCR data of SND1 expression in 10 paired CRC and adjacent non-tumor tissues. **E.** Representative IHC images of SND1 protein expression are shown (left). Original magnification, × 400. And the difference in miR-361-5p levels between SND1(Low) and SND1(High) patients with CRC (*n* = 60). The results are presented as means ± SD (*n* = 3 for each panel). Statistical significance was concluded at *P < 0.05, **P < 0.01, ***P < 0.001; # represents no statistical significance.

To further evaluate the function of miR-361-5p, it is important to determine an mRNA target of miR-361-5p that might mediate the role of miR-361-5p in tumorigenesis and invasion. The target prediction programs miRBase and TargetScan were used to predict the possible miR-361-5p targets. SND1 attracted our attention because its 3′-UTR contains a putative target sequence for miR-361-5p (Figure 4A), and SND1 is closely involved in cancer cell migration and invasion [12]. To confirm that SND1 was directly inhibited by miR-361-5p, a dual-luciferase reporter system was used. It was found that co-expression of miR-361-5p markedly inhibited the firefly luciferase reporter activity of the wild-type SND1 3′-UTR, but did not change the activity of the mutant 3′-UTR constructs (Figure 4A). The result suggested that miR-361-5p inhibited SND1 expression via binding to the 3′-UTR of SND1. Therefore, SND1 mRNA and protein expression in miR-361-5p-overexpressing or miR-361-5p-downregulated cells were investigated respectively. The overexpression of miR-361-5p decreased the endogenous expression of SND1 mRNA and protein, whereas anti-miR-361-5p inhibitor increased the expression of SND1 mRNA and protein (Figure 4B). The correlation between miR-361-5p and SND1 in 10 paired clinical CRC samples was also investigated using quantitative PCR. As expected, miR-361-5p expression was inversely correlated with SND1 mRNA expression in the 10 CRC samples (*r* = −0.581, *P* = 0.023; Figure 4C). The level of SND1 mRNA was found to be higher in cancer tissues than in corresponding non-cancer tissue in which the level of miR-361-5p is high (Figure 4D). In addition, immunohistochemical staining showed that miR-361-5p expression was significantly lower in the CRC tissues with positive SND1 expression than those with negative SND1 expression, suggesting an inverse relationship between miR-361-5p expression and SND1 expression (Figure 4E).

### SND1 mediates the tumor-suppressive function of miR-361-5p in CRC

**Figure 5:**
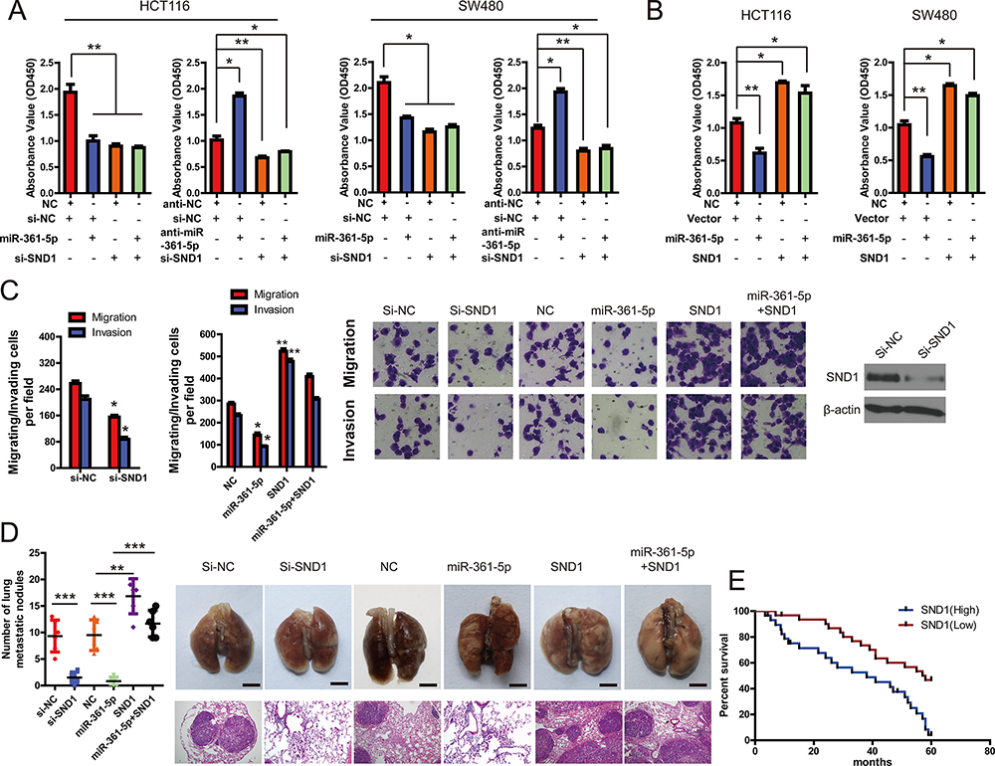
SND1 critically contributes to the cancer-inhibitory function of miR-361-5p **A.** knockdown of SND1 by siRNA markedly inhibited CRC and GC cell proliferation, and miR-361-5p overexpression did not block cell proliferation in SND1-depleted HCT116 or SW480 cells. However, miR-361-5p knockdown increased cell growth but did not promote cell proliferation in SND1-depleted HCT116 or SW480 cells. **B.** upregulation of SND1 promoted cell growth and abrogated miR-361-5p–induced growth inhibition in HCT116 or SW480 cells. **C.** Invasion assays of CRC and GC cells transfected with the vectors described in (A) and (B). **D.** Tumor metastasis analysis. the number of lung metastatic nodules in nude mice (*n* = 10 per group, left panel). A representative images and Hematoxylin and Eosin staining of metastatic tumors and normal lung tissues (right panel). The results are presented as means ± SD (*n* = 3 for each panel). Statistical significance was concluded at *P < 0.05, **P < 0.01, ***P < 0.001; # represents no statistical significance. **E.** Impact of SND1 expression on OS in patients with CRCs. Kaplan-Meier survival analysis for CRC patients according to SND1 protein expression, as determined with immunohistochemistry.

Next, we performed a series of restoration assays using HCT116 and SW480 cells to explore the functional significance of SND1 in the miR-361-5p-induced phenotype. siRNA and a construct containing the SND1 ORF were respectively used to decrease or increase SND1 expression in CRC cells. As shown in Figure 5A, on the one hand, siRNA-mediated SND1 silencing could phenocopy the proliferation-repressing effect of miR-361-5p, whereas anti-miR-361-5p could not restore cell proliferation in SND1-depleted CRC cells. On the other hand, SND1 overexpression significantly abrogated the inhibitory effect of miR-361-5p on cell proliferation (Figure 5B), suggesting that miR-361-5p exerts tumor-suppressive function in CRC via directly binding to SND1.

We went on to investigate that miR-361-5p inhibits CRC and GC cell invasion via biding to SND1. As shown in Figure 5C and 5D, silencing SND1 in CRC cells significantly decreased *in vitro* cell invasion and *in vivo* tumor metastasis, which was similar to the phenotype induced by miR-361-5p. In contrast, the ectopic expression of SND1 vector that encoded the entire coding sequence of SND1 without its 3′-UTR markedly abrogated the tumor-suppressive effect induced by miR-361-5p (Figure 5C and 5D). So it strongly demonstrated that SND1 is a functional target of miR-361-5p.

Finally, to confirm the clinical relevance of SND1 expression with tumor progression, we evaluated the prognosis of patients with different levels of SND1 expression. It was found that patients with higher SND1 expression had significantly shorter survival times than those with lower SND1 expression, with an HR of 3.11 (95% CI, 1.618 to 5.980; Figure 5E).

### SND1 suppresses the expression of miR-361-5p

**Figure 6:**
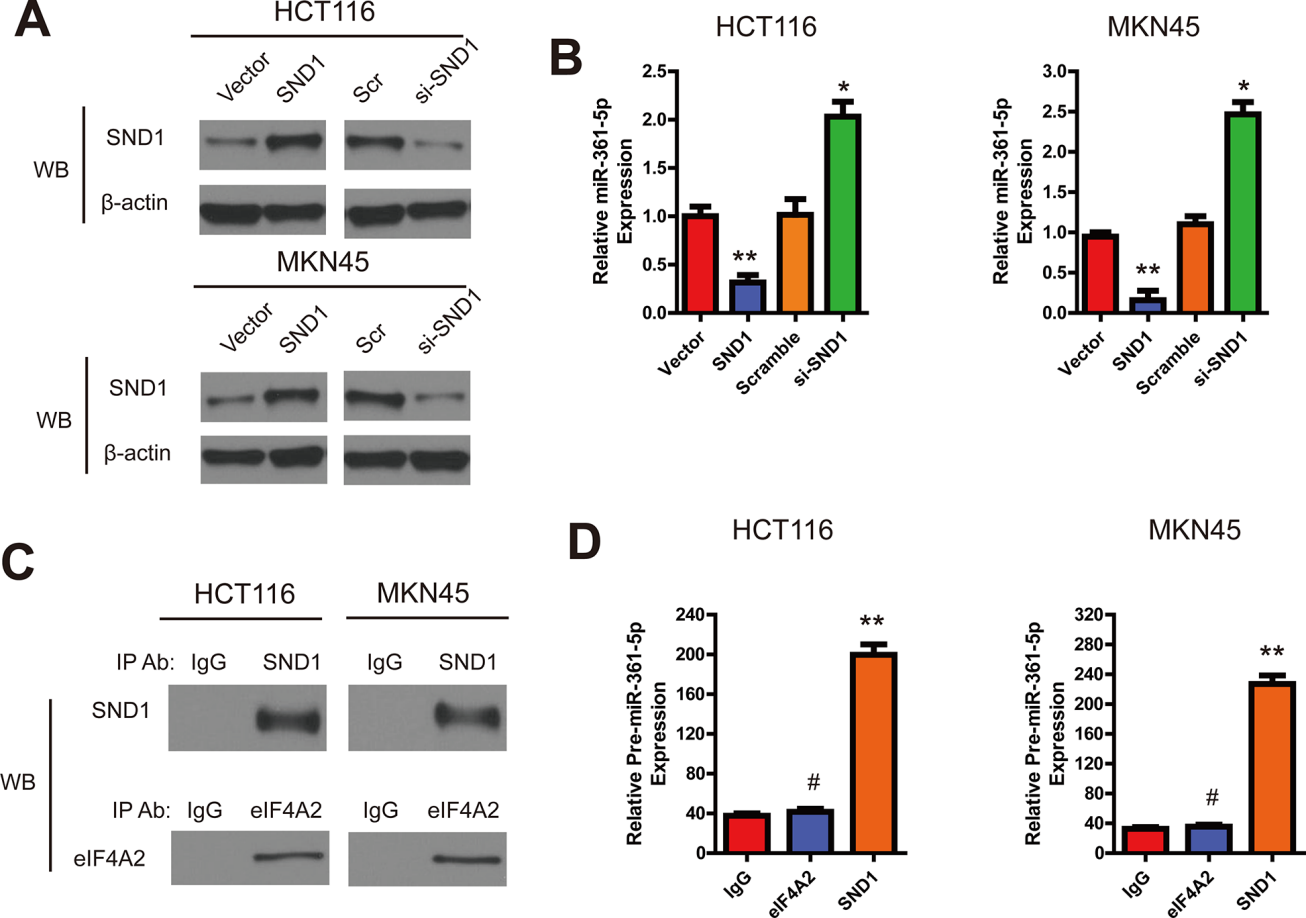
SND1 downregulates the miR-361-5p expression in CRC and GC cells **A.** The SND1 protein expression in indicated cell lysates and anti-β-actin antibody as control. **B.** The expression of miR-361-5p in indicated cells was evaluated by RT-PCR. **C.** RNA immunoprecipitation, SND1 and EIF4A2 (as a control) were specifically precipitated with the respective antibodies. IgG served as a negative control. **D.** qRT-PCR analysis of bound pre-miR-361-5p in SND1 and EIF4A2 RIPs. IgG was used as controls. The results are presented as means ± SD (*n* = 3 for each panel). Statistical significance was concluded at *P < 0.05, **P < 0.01, ***P < 0.001; # represents no statistical significance.

Because SND1 is one of the essential components of RISC which takes part in gene silencing and inhibits miRNA production [13], we next examined the effects of SND1 on miR-361-5p expression in HCT116 and MKN45 cells. The pre-miR361-5p plasmid (GFP-pre-miR-361-5p) was first transfected into HCT116 and MKN45 cells for 48 hours, the cells were then transfected with pSG5-SND1-Flag, pSG5 plasmid, SND1 siRNA, or scramble control, respectively. After 48 hours, we collected and analyzed total cell lysate or RNA from different transfected cells. As shown in Figure 6A and 6B, the endogenous SND1 was efficiently depleted by siRNA, whereas the protein level of endogenous β-actin from different lysates were comparable. Interestingly, we found that upregulation of SND1 decreased the expression of miR-361-5p, while depletion of SND1 increased the expression of miR-361-5p to 2.1-fold higher than the control group.

SND1 is known to degrade edited primary miR transcripts and precursors [14]. In order to elucidate how SND1 inhibited the expression of miR-361-5p, we performed RIP experiments to precipitate SND1-bound RNAs. Given that EIF4A2 was shown to bind to the mature miRNA, not pri-miRNA, thus, EIF4A2 RIPs were used as controls [15]. Western blotting confirmed that both proteins could specifically be precipitated (Figure 6C). However, only SND1, not EIF4A2, was confirmed as a protein binding with pre-miR-361-5p (Figure 6D). These data further indicated that SND1 could decrease the expression of miR-361-5p by forming SND1-pre-miR-361-5p complex, and consequently, exerted a negative feedback loop.

## DISCUSSION

Tumor invasion, metastatic dissemination, disease relapse, and drug resistance have been identified as classical hallmarks of cancer malignancy, and are major causes of a poor clinical outcome in cancer patients [16–17]. The present study is the first to demonstrate that miR-361-5p functions as a tumor-suppressive miRNA through directly binding to SND1 in CRC and GC. We confirmed this finding by providing the following evidence. 1) miR-361-5p was down-regulated in CRC and GC cells and tissues in comparison to the controls. 2) the expression levels of miR-361-5p exhibited a strongly negative association with lung metastasis and prognosis in clinical CRC patients. 3) Overexpression of miR-361-5p markedly suppressed cell proliferation and invasion of cancer cells, which was partially rescued by SND1. 4) SND1 was identified as a potential target of miR-361-5p in bioinformatics analysis and luciferase reporter assays. 5) SND1 bounded with pre-miR-361-5p and suppressed the expression of miR-361-5p, thus exerting a feedback loop. 6) *In vivo* studies showed that miR-361-5p significantly inhibited xenograft growth and invasion of tumors in nude mice.7), patients with higher SND1 expression had significantly shorter survival times than those with lower SND1 expression. Therefore, miR-361-5p may be highlighted as a novel agent for the treatment of patients with CRC and GC.

GC accounts for many cancer-associated deaths worldwide and is regarded as one of the most common in the world [18]. The development of GC is characterized by multi-factorial and multistage process, and *H. pylori* have been shown to the leading cause of GC [19]. It is well-known that environmental factors, including diet, are critically associated with gastric cancer risk. However, recent findings have highlighted a significantly regulatory role of the genetic background in *H. pylori* infection and GC susceptibility [20]. Among the mediators induced in response to the *H. Pylori* infection, miRNA have the potential to play a vital effect on the outcome of the bacteria-host interaction. Recently, miRNA was found to be a potential bridge from *H. pylori* infection to chronic gastritis and GC [21]. Regarding to miR-361-5p, we hypothesized that it may play the versatility role in *H. pylori* infection and GC, however, it needs to be further investigated.

Emerging evidence has indicated that miRNAs are closely involved in the regulation of various biological and pathological processes, including tumor growth, cell invasion, and tumor metastasis [16, 22]. And it has been demonstrated that many miRNAs are tumor suppressors which could tightly reduce the expression of many vital oncogenes [23]. MiR-361-5p, which is known as a tumor-suppressor miRNA, is down-regulated in human tumors and plays functional roles in several aspects of tumor development and progression [7, 24]. Interestingly, it can also function as an oncogene to facilitate cervical cancer progression [25]. However, very little was known about its role and molecular mechanism in CRC and GC. In our study, we found that miR-361-5p was down-regulated in tumor tissues and cultured cells, increased cellular growth and invasion, and *vice versa*. And its overexpression was negatively correlated with the SND1 expression and the status of tumor metastasis. Importantly, we also demonstrated that miR-361-5p is a downstream effector of SND1 because our interference experiments confirmed that miR-361-5p expression could be decreased by SND1 over-expression. Based on these data, miR-361-5p could be a tumor suppressor miRNA in CRC and GC. These findings also provide an additional molecular mechanism of the pathophysiological functions of miR-361-5p and SND1.

Another important discovery in this study is the forming of SND1-pre-miR-361-5p complex. SND1 is known as a component of the RNA-induced silencing complex [12] and it not only accelerates the kinetics of spliceosome assembly, but also enhances splicing activity [13]. Moreover, SND1 was shown to control the degradation of edited miRNAs primary transcripts and precursors, which can be promoted by tissue hypoxia [26]. This may result in a higher degradation of miRs by SND1, which is prevented by SND1 silencing [15]. Consistent with previous studies, the present study indicates that SND1 is additionally involved in the processing of the precursors to the mature miRs of the miR-361-5p. Reduction of miR processing was prevented when SND1 was silenced, indicating that SND1 is involved in the impairment of miR-361-5p maturation. This function was particularly prominent for CRC and GC tumorigenesis. We provided evidence that the multifunctional protein SND1 directly binds to the pre-miR-361-5p and reduces the expression of miR-361-5p.

## MATERIALS AND METHODS

### Cell culture

Human CRC and GC cell lines, including HCT116, HT29, LoVo, SW480, SW620, MKN45, BGC-823, HGC-27, AG5 and SGC-7901 were incubated under the conditions recommended by ATCC. All of the media (Hyclone) were supplemented with 10% FBS (Sigma).

### Cell proliferation and clonogenicity assays

The cell proliferation assay was performed with the Cell Counting Kit-8 (CCK-8, Sigma) according to the manufacturer's instructions. For the colony formation assay, 1, 000 cells were placed in each well of a 10 cm plate, transfected and incubated for 14 days, then fixed with methanol and stained with with crystal violet in 20% methanol for 20 minutes. The number of colonies was counted using an inverted microscope.

### Cell cycle analysis

Using flow cytometry with the use of propidium iodide (catalog number P-4170, Sigma) staining (5 μg/ml), cell cycle analysis was performed after 48 h of miRNA transfection. Briefly, 2 × 10^6^ cells were trypsinized, resuspended in chilled ethanol, and kept at 4°C for 15 min. Cells were then centrifuged and resuspended in 450 μl of PBS and 50 μl of RNase A (2 mg/ml), and incubated at 37°C for 30 min. 500 μl of propidium iodide was added, and cells were incubated at room temperature for 60 min in the dark. Cell cycle analysis was performed using FACScaliber Flow Cytometer (BD Biosciences) equipped with Cell Quest 3.3 software.

### Transfection

miR-361-5p mimics, scrambled miR-control, SND1 siRNA, or siRNA negative control were purchased from GenePharma (China), which were designed and chemically synthesised based on the following sequences: hsa-miR-361-5p mimics: 5′-ACGCCUGGAGAUUCUGAUAAUU-3′, negative control (NC): 5′-UUCUCCGAACGUGUCACGUTT-3′; SND1-specific siRNA: 5′-AAGGCATGAGAGCTAAT AATC-3′ (sense), 5′-AAGGAGCGATCTGCTAGCTAC-3′ (anti-sense), NC: 5′-UUCUCCGAACGUGUCACGUTT-3′ (sense), 5′-ACGUGACACGUUCGGAGAATT-3′ (anti-sense). SND1 Human cDNA ORF Clone was purchased from Origene (Origene Technologies). The lentiviral expression vector expressing miR-361-5p precursor sequence was constructed using the following primer pairs: forward: 5′-GTGGGCATATgTgACCATCA-3′; reverse: 5′-TGAgCTCAACCATACCAggA-3′. Transfections were performed using Lipofectamine 2000 (Invitrogen) at a final concentration of 100 nM, according to the manufacturer's protocol (Invitrogen). After 24 h of transfection, cells were kept in a culture medium containing 2% FBS up to 48 h. The cells were then harvested and used.

### Migration and invasion assay

6.5-mm diameter Boyden chambers with pore size of 8.0 μm (Corning) was used for migration assays. Briefly, the stable monoclonal cell lines (2.0 × 10^5^ cells per well) were resuspended in the migration medium without FBS and placed in the upper compartment of transwell chambers coated with fibronectin on the lower surface. The lower compartment was filled with 500 μl medium containing 10% FBS as a chemoattractant. Cells were fixed in 4% formaldehyde and stained with 0.1% crystal violet after 24 hours. Ten random fields were counted under a light microscope (Carl Zeiss, Germany). However, cell invasion (2.0 × 10^5^ cells per well) was evaluated in 24-well matrigel-coated invasion chambers after 48 hours.

### Wound healing assays

To measure cell migration, CRC and GC cells were plated into 35 mm dishes and cultured for 1 day. A scraped line was created with a 200 ul pipette tip once the cells nearly achieved 100% confluence. Then we replaced the medium and cultured the cells for 24 hrs. The speed of wound closure was imaged with an inverted microscope TE-2000S (Nikon) and the rate of closure was assessed.

### RNA isolation and RT-PCR

Total RNA was extracted with Trizol reagent (Invitrogen) and a reverse transcription was performed, with U6 small nuclear RNA as an internal normalized reference. The Mir-VanaTM miRNA isolation kit (Ambion) was used to isolate total RNA including microRNA from cell lines and frozen patient samples according to the manufacturer's protocol. For miRNA detection, 100 ng total RNA was reverse transcribed into cDNA using specific primers designed for miRNA analysis. RNA was measured with an RNA 6000 Nano Assay kit and 2100 Bioanalyzer (Agilent Technologies, USA). The RNA was used to prepare cDNA by using the iScript cDNA Synthesis Kit (Bio-RAD) according to the manufacturer's instructions. RT-PCR was performed using Platinum Taq DNA Polymerase (Invitrogen) with human specific primers for miR-361-5p: Forward primer: ATAAAGTGCTGACAGTGCAGATAGTG, miR-361-5p Reverse primer: TCAAGTACCCACAGTGCGGT, and U6 forward primer: CTCGCTTCGGCAGCACA, U 6 reverse primer: AACGCTTCACG AATTTGCGT. RT:5′-gTCgTATCCAgTgCAgggTCCgAggTATTCgCACTggATAC-3′. The RT-PCR conditions were as follows: Stage I: 95°C for 3 min, 53°C for 1 min, 72°C for 30 sec (2 cycles); Stage II: 95°C for 3 min, 53°C for 1 min, 72°C for 30 sec (55 cycles); Stage III: 72°C for 5 min. The PCR product was run on a 2.5% agarose gel prepared in 1x Tris-acetate EDTA buffer containing ethidium bromide and analyzed using a Gel-Doc apparatus.

### RNA immunoprecipitation

The MagnaRIP™ Kit was used to perform RIP according to the manufacturer's protocol (Millipore) with antibodies against SND1 (Santa Cruz), EIF4A2 (Santa CruZ) or IgG as negative control. Briefly, CRC and GC cells were treated with 0.1% formaldehyde in PBS, for 30 min at 4°C and subsequently lysed with RIP lysis buffer for 40 min on ice. Antibody coupled beads were incubated with CRC cell lysates for 24 hours at 4°C. The RNA was purified with phenol–chloroform, ethanol precipitated, and resuspended in water.

### Dual-luciferase 3′ UTR-reporter assay

Dual-luciferase 3′ UTR reporter assay was carried out to validate SND1 as a direct target of miR-361-5p. Wild-type (WT) or mutant of 3′ untranslated region (UTR) sequences of SND1 were inserted into the Fse I and Xba I sites of the pGL3 vector (GeneChem). HEK293 cells infected with anti-miR-361-5p lentivirus or negative control (NC) lentivirus were plated into 96-well plates. pGL3 vector (40 ng) with the above sequence was cotransfected with 10 ng of pRL-TK vector into cells by Lipofectamine LTX (Invitrogen). Twenty-four hours later, cells were collected according to the manufacturer's protocol (Promega) and firefly and Renilla luciferase activity was detected using Dual-luciferase Reporter Assay System Kits (Promega) with a Victor X machine (PerkinElmer). Additionally, a mutant SND1 3′ UTR reporter construct was made by site-directed mutagenesis in the putative target site of miR-361-5p using Quickchange XL site-directed mutagenesis kit (Agilent Technologies). All PCR products were verified by DNA sequencing. The normalized luciferase activity was expressed as a ratio of firefly luciferase to Renilla luciferase units.

### Confocal microscopy

CRC and GC cells were respectively fixed and permeabilized with 4% formaldehyde, 0.2% Triton X-100 in PEM buffer (5 mm EGTA, 2 mm MgCl_2_, 100 mm PIPES, pH 6.8) for 15 min at room temperature and thereafter postfixed with methanol for 10 min at −20°C. Cells were stained using indicated antibodies, Alexa 488 anti-mouse and Texas Red anti-rabbit secondary antibodies (Molecular Probes). Confocal images were taken using LSM510 program and Zeiss confocal microscope with an Argon laser (488 nm) and HeNe laser (543 nm) and *a* × 63 objective. Green emission was detected using a 505-nm low pass filter and red emission using a 630-nm low pass filter.

### *In situ* hybridization

In order to detect the expression of miR-361-5p in patients' tissues, *in situ* hybridization technique was used using Biochain kit (catalog#: K2191050; Biochain IsHyb In Situ hybridization kit) according to the manufacturer's protocol (Biochain and EXIQON). Briefly, tissues were deparaffinized and fixed in 4% paraformaldehyde in DEPC-PBS for 20 min and digested with 0.1% Triton-X and 2X standard saline citrate for 25 min. The tissue were prehybridized with prehydridization solution, incubated with hybridization buffer and digoxigenin labelled probe (EXIQON) for 24 hours. This followed the subsequent incubation of tissues overnight with the AP-conjugated anti-digoxingenin antibody. Further, the slides were washed for 5 min with 1X Alkaline Phosphatase buffer twice and visualised with NBT/BCIP (Pierce) followed by nuclear fast red counterstaining. The slides were mounted and analyzed under microscope.

### Western blot

Cells were transfected with miR-361-5p mimics, NC in presence or absence of miR-361-5p inhibitor. Treated cells were washed with ice-cold PBS and then lysed by a cell lysis buffer (Pierce). Cell protein lysates were separated in 8% SDS denatured polyacrylamide gel and then transferred onto a polyvinylidene difluoride membrane. The membranes were blocked with 5% nonfat dry milk in Tris-buffered saline, pH 7.4, containing 0.05% Tween 20, and were incubated with primary antibodies for SND1(Abcam), β-actin, vimentin, anti-E-cadherin (Santa Cruz) and GAPDH (Cell Signaling) at 4°C overnight respectively. Membranes were washed and incubated with horseradish peroxidase-conjugated secondary antibodies (BIO-RAD) according to the manufacturer's instructions. The protein of interest was visualized using ECL Western blotting substrate (Pierce).

### Immunohistochemistry

The paraffin-embedded tissue samples from postoperative patients were deparaffinized in xylene, rehydrated and blocked with 10% goat serum before incubating with primary antibody. The samples were incubated overnight with a primary antibody, and subsequent secondary antibody followed by DAB or fluorescence labeled secondary antibody. The slides were visualized using a microscope or inverted fluorescence microscope TE-2000S (Nikon). Actin tracker green, DAPI and fluorescence labeled secondary antibody were purchased from Molecular Probe.

### *In vivo* tumorigenic assay

All animal experiments were performed in accordance with NIH guidelines for the use of experimental animals. Male Nu/Nu mice, obtained from the Experimental Animal Center of Shanghai Institute for Biological Sciences (SIBS), were injected subcutaneously with 1.5 × 10^6^ miR-361-5p or NC or anti-miR-361-5p or SND1 or SND1-siRNA or its control stable monoclonal cells to establish a CRC xenograft model. After four weeks, eight mice were sacrificed and subcutaneous tumors were collected for analysis of the expression of *SND1* and miR-361-5p. After 8 weeks, the remaining mice were sacrificed, and lungs were isolated for examination of the number of metastatic tumors. Tumor volume (V) was estimated from the length, width (w), and height (h) of the tumor. For each specimen, haematoxylin and eosin (HE) staining was performed.

### Patients and tissue specimens

From January 2008 to May 2014, a total of 60 pairs of CRC and non-tumor samples were gathered from patients who had CRC resection performed in Hongqi Hospital of Mudanjiang Medical University. The samples were used for subsequent RNA extraction or immunohistochemistry (IHC) or immunofluorescence (IF). All human materials were obtained with informed consent and approved by the ethics committee of Hongqi Hospital of Mudanjiang Medical University.

### Statistical analysis

Student's t test or the Chi square test was applied to analyze the differences between groups. The relationship between the miR-361-5p level and SND1 expression was analyzed using Pearson's correlation. Statistical analysis was performed with SPSS software (version 17). *P* < 0.05 was considered statistically significant. The results are expressed as mean ± standard deviation (S.D.) from at least three independent experiments. Statistically significant data are indicated by asterisks *P* < 0.05 (*), *P* < 0.01(**), *P* < 0.01(***).

## CONCLUSIONS

miR-361-5p overexpression inhibited tumor growth and metastasis in CRC and GC cells through the down-regulation of SND1. In turn, SND1 activation significantly caused miR-361-5p down-regulation. Thus, the miR-361-5p/SND1 feedback loop provides a new avenue to understand the mechanism of the tumor invasion and metastasis in CRC and GC.

## Abbreviations

miRs: MicroRNAs
CRC: colorectal carcinoma
GC: gastric cancer
miR-361-5p: microRNA-361–5p
SND1: Tudor staphylococcal nuclease
EBNA2: Epstein-Barr virus nuclear antigen 2
PC1: polycystin-1
